# Sex Differences in Insomnia and Circadian Rhythm Disorders: A Systematic Review

**DOI:** 10.3390/medicina60030474

**Published:** 2024-03-13

**Authors:** Evelina Pajėdienė, Viltė Urbonavičiūtė, Vita Ramanauskaitė, Lukas Strazdauskas, Ambra Stefani

**Affiliations:** 1 Department of Neurology, Lithuanian University of Health Sciences, Eivenių g. 2, 50161 Kaunas, Lithuania; 2 Faculty of Medicine, Lithuanian University of Health Sciences, A. Mickevičiaus g. 9, 44307 Kaunas, Lithuania; vita.ramanauskaite@stud.lsmu.lt (V.R.); lukas.strazdauskas@stud.lsmu.lt (L.S.); 3 Department of Neurology, Medical University of Innsbruck, Anichstraße 35, 6020 Innsbruck, Austria

**Keywords:** insomnia, circadian rhythm disorders, sex differences

## Abstract

Insomnia and circadian rhythm disorders are increasingly common in modern society and lead to significant challenges for people’s health and well-being. Some studies suggests that men and women differ in neurohormonal secretion, biological processes, and brain morphology. Thus, such differences may affect the etiology, manifestation, and course of sleep disorders, including insomnia and circadian rhythm. This systematic review aims to synthesize the existing literature on sex differences in insomnia and circadian rhythm disorders. PubMed, MEDLINE, Epistemonikos, and Cochrane databases were searched for articles published from inception until 5 September 2023, not older than five years. We performed a systematic search using MESH and non-MESH queries: (sex differences) or (male and female differences) or (men and women differences) or (men and women) AND (insomnia) or (sleep wake disorder*) or (sleep wake rhythm disorder*) or (circadian rhythm disorder*) or (sleep cycle disruption) or (sleep cycle disorder*). Out off 2833 articles screened, 11 studies were included. The prevalence of insomnia is higher among women, and their sleep is more regular and stable compared to men. Studies evaluating the impact of the stressful situation associated with the lockdown on women’s and men’s insomnia present discordant results concerning sex differences. Women’s circadian rhythm was found to be more stable and less fragmented than men’s. However, the progression of peak activity time with age was more pronounced in men. The current literature suggests that risk factors for insomnia and circadian rhythm disorders affect men and women differently. These include cerebrovascular and cardiometabolic factors, shift work, and infections. The long-term effects of insomnia seem to be more relevant for the male sex, shortening lifespan more than in women. By summarizing and analyzing existing studies, we highlight the need for further research to improve understanding of the interaction between sex and sleep.

## 1. Introduction

As one of the main symptoms of sleep-related disturbances, insomnia is a common clinical condition characterized by difficulty in initiating and/or maintaining sleep [1], with relevant health consequences. In fact, sleep is necessary for preserving both physical and mental health as well as brain health [2]. Insomnia can appear alone or in conjunction with other medical diseases or mental health disorders, and if left untreated, it can increase the chance of developing and exacerbating any of these conditions [3]. Roughly 10% of adults face a chronic insomnia disorder, and another 20% deal with short-term insomnia symptoms. Insomnia commonly becomes a persistent condition, with a 40% continuity rate over a 5-year period [1].

The circadian timing system works in conjunction with a circadian rhythm homeostatic process to determine the timing, length, and consolidation of sleep [4]. Persistent or recurrent misalignments between the circadian rhythm pattern and the social clock indicate the occurrence of circadian rhythm disorders, which result in impaired daytime function and excessive daytime sleepiness or insomnia [5]. Sleep is regulated by several regions of the brainstem, hypothalamus, thalamus, and forebrain [6], and the suprachiasmatic nucleus is where the body’s internal clock is found [5].

Physiological sleep patterns might vary between the sexes, potentially reflecting variability in sleep disorders’ risk and symptoms [7]. Men and women exhibit differences in body and brain morphology, biological processes, and neurohormone secretion, which can influence the expression and regulation of behavioral and physiologic functions, including sleep and circadian rhythms [8]. As a result, there may be differences in the etiology of insomnia, its clinical manifestations, and the patient’s attitude toward and coping with insomnia between the sexes [9]. Previous epidemiological research has shown that women are more prone than men to experience insomnia symptoms [10]. Biological variables tend to interact with psychosocial factors, further contributing to differences in sleep and/or circadian rhythms and susceptibility to the development of sleep disorders between women and men [8]. However, there are relatively few studies investigating the differences between the sexes in relation to manifestations of insomnia and circadian rhythm disorders [7].

The aim of this review is to provide an overview of the existing literature about the impact of sex differences on insomnia and circadian rhythm disorders and suggest future directions to improve knowledge in this field and sex-specific management of these sleep disorders.

## 2. Materials and Methods

### 2.1. Search Strategy

**Figure 1:**
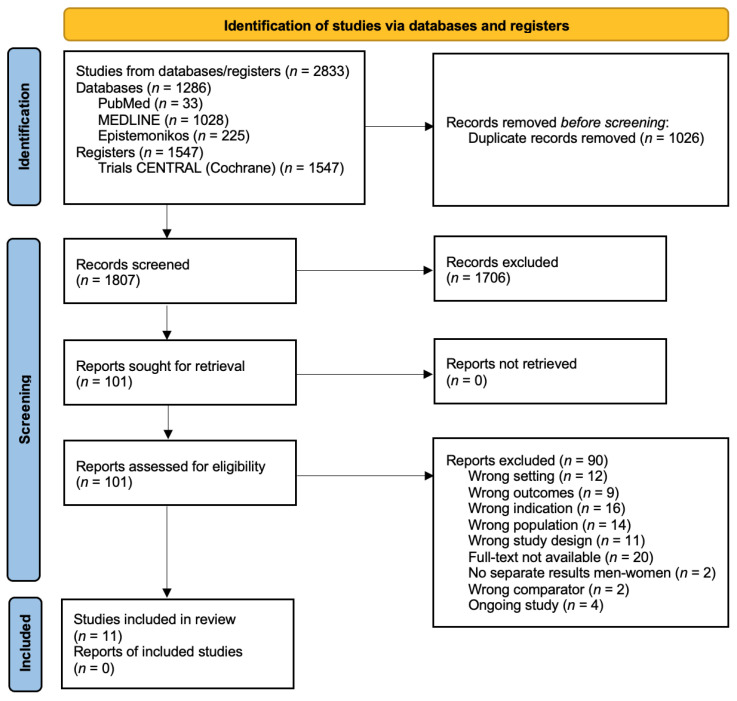
PRISMA 2020 flow diagram for the identification of the studies included in the systematic review.

This systematic review has been registered with the international prospective register of systematic reviews PROSPERO, submission ID: CRD42023477828, and was performed in agreement with the Preferred Reporting Items for Systematic Reviews and Meta-Analyses Protocols (PRISMA-P) [11]. The PRISMA 2020 flow diagram is shown in Figure 1. (PRISMA 2020 flow diagram for the identification of the studies included in the systematic review). This study satisfied all the recommended items reported by the PRISMA-P checklist. A comprehensive literature search was performed for studies assessing potential sex differences in insomnia and circadian rhythm disorders.

In this regard, as previously reported, the PICO (Patient Problem, Intervention, Comparison, and Outcome) framework was applied to develop the literature search strategy. The “patient problem” included adult patients suffering from insomnia or circadian rhythm disorders; “intervention” and “comparison” were not included; the “outcome” assessed was any sex-related difference in the symptoms, etiology, distribution, prevalence of insomnia, and circadian rhythm disorders.

The literature search was run from inception until 5 September 2023. No language restrictions were applied. Three independent researchers performed a systematic search of PubMed, MEDLINE, Epistemonikos, and Cochrane databases to identify relevant studies. Furthermore, we hand-searched the references of included articles for additional studies to screen. We performed a systematic search using MESH and non-MESH queries: (sex differences) or (male and female differences) or (men and women differences) or (men and women) AND (insomnia) or (sleep wake disorder*) or (sleep wake rhythm disorder*) or (circadian rhythm disorder*) or (sleep cycle disruption) or (sleep cycle disorder*).

Search results were exported to Covidence and analyzed. Covidence streamlines evidence synthesis with a gold standard process for creating high-quality systematic reviews. Duplicates were eliminated using the RefWorks function. Subsequently, three authors screened all the literature search results; first by title and abstracts according to the eligibility criteria. Later, the reviewers analyzed the full texts of the articles for relevance to the literature review.

### 2.2. Eligibility Criteria

We included studies with insomnia or circadian rhythm disorder patients aged ≥18 years. Study types included were: (1) cohort studies (CS), (2) case-control studies (CCS), (3) cross-sectional studies (CSS), (4) randomized clinical trials (RCTs), (5) non-randomized clinical trials (non-RCTs). The included studies were no older than five years. Studies excluded were: (1) case reports, (2) case series, (3) reviews, and (4) meta-analyses. Single-sex studies were excluded. Animal studies were not included.

### 2.3. Measured Outcomes

Sex differences are the main outcome.

### 2.4. Assessment of Risk of Bias

Quality assessment of the studies was conducted independently by three reviewers, who screened the retrieved studies, extracted and synthesized the included studies’ information, and assessed their quality ranking. Discrepancies were resolved via a team discussion with two reviewers. The methodological quality of observational cross-sectional and longitudinal studies was evaluated using the JBI Critical Appraisal Checklist Tool [12] for analytical cross-sectional studies. Specifically, this assessment tool classifies studies as having “low” (0~2 “yes”), “moderate” (3~4 “yes”), or “high” (>5 “yes”) methodological quality. The checklist consisted of eight question items assessing the inclusion criteria for the definition and detailed description of the sample, use of valid and reliable ways to measure the exposure, use of objective and standard criteria to measure the condition, identification, and strategies to deal with confounding factors, use of valid and reliable ways to measure outcomes, and suitability of statistical analyses. The Critical Appraisal Skills Program (CASP) [13] checklist was used for cohort studies. The CASP checklist consists of 12 questions, which are divided into three subgroups: A (are the results of the study valid?), B (what are the results?), and C (will the results help, locally?). Studies were classified according to their quality assessment percentage, as follows: ‘high quality’, >85%; ‘moderate to high’, 75–85%; ‘moderate’, 60–75%; ‘low to moderate’, 50–60%; and ‘low’, <50%.

### 2.5. Statistical Analysis

Data from the original papers were extracted and reported via qualitative synthesis.

## 3. Results

### 3.1. Study Selection and Characteristics

Eleven studies were included in the systematic review, and the PRISMA flowchart is shown in Figure 1**.** (PRISMA 2020 flow diagram for the identification of the studies included in the systematic review). Information about these 11 studies is summarized in terms of author(s), years, country, study design, aims, population, and main findings in Table 1. Among them, seven were cross-sectional studies, one was a longitudinal study, and three were cohort studies. In conducting the literature search, we did not identify any Randomized Controlled Trials (RCTs) on this topic. This could be attributed to the fact that, during our selected time period, the COVID-19 pandemic may have impacted the conduction of RCT studies. The included studies represent a diverse population from eleven countries, with six studies conducted in Europe (Sweden, Greece, Portugal, Italy, Poland, and Spain), and one study from each of the following countries: USA, Australia, Korea, Brazil, and China. Thus, despite being diverse, most of the included studies investigated a White or Asian population. The total number of participants represented in this review was 344,958 from eleven studies, with a sample size ranging from 157 to 308,683. The proportion of women in all included studies ranged from 40.7% to 60.5%. The mean age ranged from 18 to 90 years across the included studies. The study samples were highly diverse, and there was significant variability in the methods used to assess insomnia and circadian rhythm disorders. Taking into account this drawback, below, we summarize the most relevant details from the included articles and the sex differences identified by the authors.

### 3.2. Quality Appraisal

**Figure 2:**
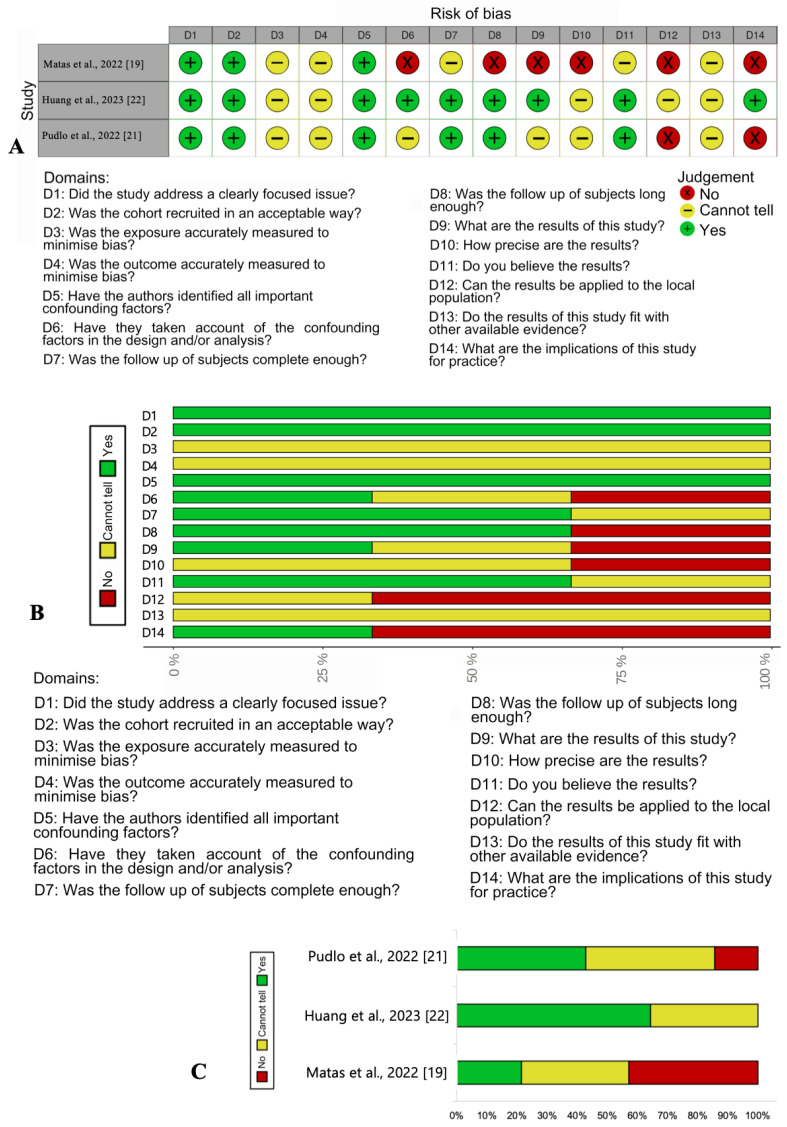
Risk of bias in cohort studies: [19,21,22]. (**A**) Traffic light plot of the summary of the authors’ judgments about risk of bias items for each included cohort study. (**B**) Risk-of-bias graph: The authors’ judgments regarding each risk-of-bias item presented as percentages across all included studies. (**C**) Risk-of-bias graph: summary of the total percentage assessment of all risk of bias elements for each study separately.

**Figure 3:**
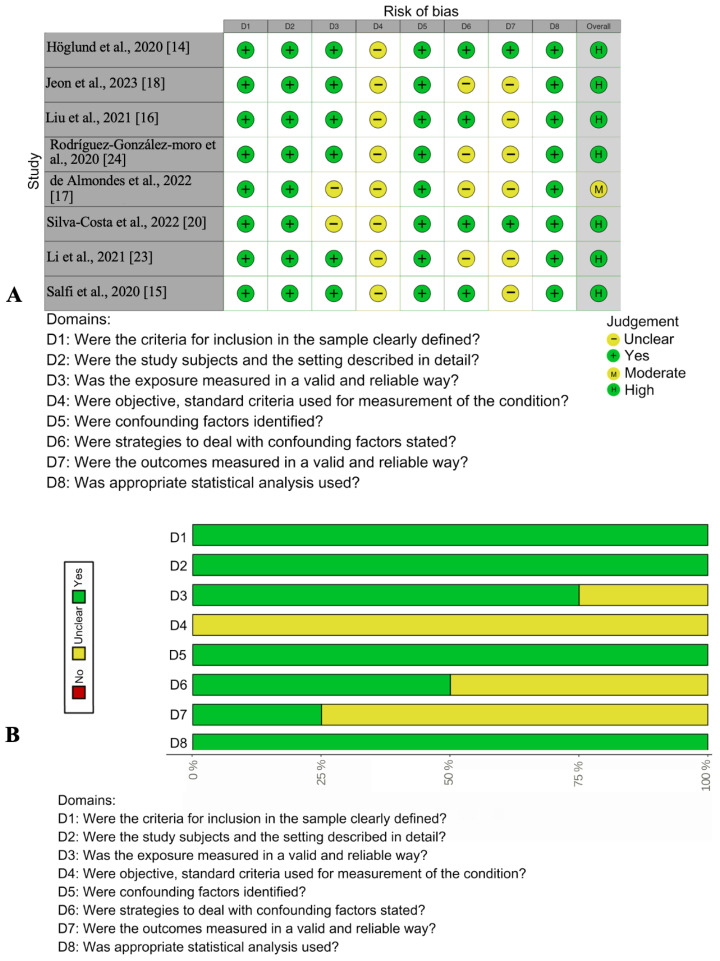
Risk of bias in cross-sectional studies: [14,15,16,17,18,20,23,24]. (**A**) Traffic light plot of the summary of the authors’ judg-ments regarding each risk-of-bias item for each included study. (**B**) Risk-of-bias graph: The authors judgments regarding each risk-of-bias item presented as percentages across all included studies.

Quality assessments are shown in Figure 2. (risk of bias in cohort studies) and Figure 3. (risk of bias in cross-sectional studies). Out of the eight included cross-sectional or longitudinal studies, seven were of high methodological quality, and one was of moderate methodological quality. Out of the three included cohort studies, one had moderate quality, and two had low moderate quality. The factors negatively impacting study quality were mainly related to implications for practice, adaptation of results to the local population, as well as some uncertainty regarding the measurement of exposure and outcome, and the use of subjective criteria.

### 3.3. Insomnia

#### 3.3.1. Prevalence

Only one study examined the prevalence of insomnia according to its severity in a sample of 3046 individuals. Insomnia was assessed by seven items of two-dimensional sleep quality and non-restorative sleep according to the Swedish version of the Karolinska Sleep Questionnaire (KSQ), and information about the presence of sleep difficulties in the past three months was collected according to DSM-5 insomnia criteria. Women tended to have a higher prevalence of insomnia than men, which decreased with age. Men in the age group of 30–39 years had the highest risk of developing insomnia and presented more severe insomnia symptoms than women; the risk was lowest in the age group of 50–69 years and started increasing again in the age group of 70–79 years. In both men and women, insomnia symptoms were milder in the age group of 50–69 years [14].

#### 3.3.2. Insomnia during the COVID-19 Lockdown

Four studies examined how the stressful situation associated with the COVID-19 lockdown affected insomnia in men and women.

One of these studies included 2701 respondents interviewed using the Pittsburgh Sleep Quality Index (PSQI) and the Insomnia Severity Index (ISI) sleep scales. Logistic regression analysis was performed to assess the sex differences in the prevalence of insomnia at the two assessment times [15]. This study showed that, whereas before the lockdown, insomnia occurred more often in women compared to men, during the lockdown, insomnia symptoms occurred more often in men.

Another study administered the PSQI scale to 2858 subjects. Insomnia occurred in 19.6% of the participants. The results revealed that during the COVID-19 outbreak, no significant sex differences in the incidence of insomnia were present. It was also found that the occurrence of insomnia was influenced by previous psychological problems, as well as changes in income and standard of living. However, this study is likely not representative of the general population, as the proportion of older respondents was lower than expected [16].

Two other studies showed opposite results. One of them focused on the elderly population, with 914 participants aged 65–90 surveyed online. Anonymous survey results were published during the 1st wave of COVID-19 (April–August 2020) in Portugal. The survey data showed that some sleep disorders worsened significantly during the first lockdown when participants answered the survey. The worsening of sleep disorders was determined using the Morbidity Index (MI) for sleep scale. Significant changes were detected for women’s insomnia, restless leg syndrome, and men’s sleep apnea. Compared to men, women reported a longer time to fall asleep, a higher frequency of awakenings during sleep, lower sleep efficiency, and poorer sleep quality [17]. In another study, 4000 participants were surveyed using a modified ISI scale and the Epworth Sleepiness Scale (ESS). Compared to previous data, insomnia increased 1.5-fold during the pandemic, and difficulties falling and staying asleep increased 3-fold and 2-fold, respectively. The prevalence of insomnia among women was significantly higher than among men in all age groups, i.e., 21 to 69 years. Among men, the highest prevalence of insomnia was found in the age group of 20–29 years, while among women, it was in the age group of 30–39 years. In both sexes, a decreasing trend with age was observed. The occurrence of insomnia was associated with night work schedule, smoking, and being single. Moderate or severe insomnia was found in 12.9% of participants. Of note, the difficulty in falling asleep and maintaining sleep among women was most pronounced in the 30-year-old age group and among men in the 60-year-old age group [18].

#### 3.3.3. Risk Factors of Insomnia

Three articles examined one or more risk factors for insomnia. A study aimed at investigating whether cerebral circulation disorders affect sleep structure. In this study, data from 157 patients with ischemic stroke were retrospectively evaluated during their hospitalization in a Portuguese stroke unit and reevaluated two years later. The use of psychotropic medication and referral for sleep counseling and/or follow-up sleep testing were recorded. Insomnia after stroke, diagnosed according to the third edition of the International Classification of Sleep Disorders (ICSD3), was present in 45 out of 157 (28.6%) patients. Male sex (*p* = 0.006) and previous minor vascular events (*p* = 0.013) were found to be significantly associated with the development of insomnia after ischemic stroke [19].

Another study analyzed the relationship between cardiometabolic factors and insomnia symptoms or sleep duration. The cross-sectional study used data from the Brazilian Longitudinal Study of Adult Health Survey, involving 7491 women and 6232 men. Administered questionnaires included questions about socioeconomic conditions, lifestyle, and sleep characteristics. 12 h fasting blood samples were also taken to determine serum cholesterol, triglycerides, and glycosylated hemoglobin. In addition, blood pressure, weight, and height were measured. 27.8% and 19.3% of women and men, respectively, reported symptoms of insomnia. The study found that sleep duration and insomnia symptoms were associated with obesity, hypertension, and increased glycosylated hemoglobin in women and with hypertriglyceridemia in men [20].

Based on evidence that most infections affect sleep patterns by reducing wake after sleep onset and diminishing REM sleep duration, another study analyzed the link between COVID-19 infection and insomnia. The Athens Insomnia Scale (AIS) was used to quantitatively measure symptoms of insomnia according to ICD-10 criteria in a sample of 200 respondents with COVID-19. A total score of 6 points or above on the AIS is indicative of a high likelihood of the presence of insomnia. A significant positive correlation was found between the development of insomnia and the duration of symptoms of infection. When comparing the AIS results of men and women, the latter scored higher. A significant sex difference was also found when comparing individual questions: women had worse rates of total sleep duration, well-being, physical and mental readiness the following day, and sleepiness during the day [21].

#### 3.3.4. Long-Term Effects of Insomnia

Analysis of the included studies revealed that insomnia had a different effect on life cardiovascular disease (CVD) life expectancy in men and women. A cohort study including 308,683 middle-aged participants used a composite sleep score to assess participants’ self-reported chronotype, sleep duration, insomnia complaints, snoring, and daytime sleepiness. The composite score was used to define three categories of sleep: poor, moderate, and healthy. Three clinical sleep disorders were also identified, as documented in primary care and inpatient records within two years prior to inclusion in the cohort study: insomnia, sleep-related respiratory problems, and other sleep disorders. Cardiovascular disease-free life expectancy was estimated using three-state Markov models conditional on survival at age 40, taking into account different sleep profiles and clinical disorders. This study showed a correlation of poor sleep with progressively lower CVD-free life expectancy in both sexes. Women in the poor sleep category had a CVD-free life expectancy of 31.46 years, while women in the healthy category had a CVD-free life expectancy of 33.26 years, corresponding to a loss of CVD-free life expectancy of 1.80 years in women with poor sleep. The results were more pronounced among men, with a 2.31-year loss of CVD-free life in the poor sleep category compared to the healthy sleep category. Results were similar for the presence of sleep disorders: men with insomnia lost 3.84 years of CVD-free life compared to those without a diagnosis of insomnia. Women with any of the assessed sleep disorders lost 1.43 years of CVD-free life expectancy compared to those without sleep disorders [22].

### 3.4. Circadian Rhythm Disorders

Several studies have investigated the relationship between rest–activity rhythm (RAR) and neurodegenerative diseases, cardiometabolic disorders, and diabetes mellitus, using accelerometer-measured data. However, research examining sex differences in circadian rhythm disorders is lacking. One study assessed sex differences in data from the US National Health and Nutrition Examination Survey. During this study, 8200 participants used an ActiGraph accelerometer at a sampling frequency of 80 Hz on their non-dominant wrist 24 h/day for seven consecutive days. Non-parametric analysis methods were used to quantify RAR from the collected data. In the study rest–activity rhythm strength index (RARSI), average activity level and peak activity time parameters were used. RARSI is a numerical measure quantifying how well-defined and consistent an individual’s daily sleep–wake cycle is. A higher RARSI indicates a strong and consistent circadian rhythm, while a lower one suggests a less organized pattern. The average activity level in circadian rhythm is the overall amount of physical activity someone has throughout a day. It helps in understanding how activity is distributed over time. Peak activity time is the most physically active time of the day, revealing insights into physiological energy peaks. Women’s average activity levels were higher than men’s, and the circadian rest–activity rhythm was found to be more stable and less fragmented in women compared to men [23].

A descriptive–observational, cross-sectional, and prospective study analyzed the relationship between different work shifts and circadian rhythm disorders. The study involved 476 public employees, who were followed for 28 months. The PSQI scale was used to assess sleep quality, and the reduced scale of the Horne and Österberg Morningness–Eveningness Questionnaire was used to analyze circadian typology or chronotype. In contrast to previous studies, no significant sex differences were found in overall sleep quality and chronotype. However, differences in sleep duration were found: women had shorter sleep durations compared to men. The dominant chronotype among participants was the morning one, with 62% [24].

## 4. Discussion

The aim of our systematic review was to examine and summarize the existing literature on sex differences in insomnia and circadian rhythm disorders in order to address future directions for improving knowledge in this field and to develop sex-specific management strategies for these sleep disorders. A detailed analysis of the included studies revealed several noteworthy findings, including a higher prevalence of insomnia in women, sex differences in the age groups mostly affected by insomnia, as well as more stable and regular rest/activity pattern in women compared to men.

Insomnia prevalence was higher among women [14], in line with the results of a recent meta-analysis including 13 articles [25]. Some authors suggested that this finding could be related to sex hormones. The main sex differences appear during the first menstrual cycle when changes in ovarian function influence the circadian cycle through estradiol and progesterone. Studies on both animals and humans suggest that progesterone reduces arousal [25,26,27], whereas estrogen seems to boost the activity of the neurotransmitter norepinephrine, leading to more time spent in REM sleep and a shorter REM latency. When female reproductive hormones decrease, melatonin production and release decrease, potentially impacting sleep [28]. As hormones affect sleep architecture, the risk of sleep disorders increases at every stage of a woman’s life, from childhood and menarche to pregnancy and menopause [29]. In line with this, it can be hypothesized that sex hormones contribute to the higher prevalence of insomnia in women compared to men.

Our review covered the period 2018–2023, which was strongly impacted by the COVID-19 pandemic. Many countries have had to take extreme measures, such as lockdown, quarantine of affected people, and social distancing, which have had significant consequences on people’s psychological state [30]. A double-blind, placebo-controlled study found that men are able to respond appropriately to stressors, even when the sympathetic nervous system (SNS) or the hypothalamic–pituitary–adrenal axis (PHA) is suppressed [31]. On the contrary, in women, suppression of the SNS or PHA resulted in increased activation of the other system, respectively, resulting in depressed mood in response to stress. In this review, we examined how insomnia, which can result from stressful events, affects men and women differently. We found conflicting results, as insomnia during the COVID-19 pandemic was found to be more common in women in two studies, in men in one, and no significant sex difference was reported in the fourth study included in this review [15,16,17,18]. Nevertheless, some limitations of the research methodology of these four studies should be considered. First, different studies focused on different age groups, i.e., younger [16] or older adults [17], and age has an impact on the prevalence of insomnia. Also, the sample sizes in the studies ranged from 914 to 4000 [15,16,17,18]. Moreover, results may have been affected by the different restrictions applied in different countries during the pandemic, in particular, considering that some of the examined studies were conducted in countries highly affected by the pandemic, i.e., where the virus began to spread, and Italy, which was the first hot spot outside China. In addition, cultural differences may have played a role.

Based on the results of our literature review, risk factors for insomnia affect men and women differently. However, we did not find a single risk factor identified by several studies, underlying the need for expanding research on sex differences and insomnia. The risk factors that we considered include very different aspects: cerebrovascular and cardiometabolic factors, infections, and shift work [19,20,21]. One of the risk factors increasing the occurrence of insomnia is ischemic stroke. According to some authors, insomnia occurs in about half of the patients in the first month after a stroke [32]. Insomnia or its symptoms are also much more common in stroke patients than in the general population [33]. As for what concerns sex differences, according to our review, post-stroke insomnia is diagnosed more often in men [19]. Such results are in line with a meta-analysis, which included 15 studies examining the prevalence of insomnia and found that, on average, 59.8% of patients with post-stroke insomnia were men. In only one study, less than half of stroke cases were men [33]. Other important risk factors for insomnia include cardiometabolic disorders. According to a multivariate analysis, patients with a high body mass index, central obesity, hypertension, hyperglycemia, and metabolic syndrome are more likely to suffer from insomnia [34]. Analyzing sex differences, we were able to determine that cardiometabolic risk factors related to insomnia in women include obesity, arterial hypertension, and increased glycosylated hemoglobin, while in men, hypertriglyceridemia represented a risk factor [20].

It should be noted that some of the included articles on insomnia analyzed COVID-19 as a stressful event and its impact on sleep. Of note, one study looked at the link between COVID-19-related infection and insomnia [21], showing that women were more affected by the inflammatory response caused by COVID-19 [21]. Other authors have also found such a trend. During a 5-year follow-up, worse subjective sleep quality was observed in women, and this was associated with an increase in IL-6 and CRP concentration [35]. In line with this, a meta-analysis of 72 studies found a significant relationship between increased levels of IL-6 and CRP with sleep disorders [36]. Thus, the reported more severe insomnia symptoms in women with COVID-19-related infection may be related to a stronger inflammatory response compared to men.

According to some authors, chronic insomnia is associated with an increased risk of CVD and mortality [37]. We were able to find a study that examined the association of poor sleep quality with decreased CVD-free life expectancy. Sex differences were noted, highlighting that men lost more years of CVD-free life than women [22]. Another study looking at the mortality risk associated with insomnia came to a similar conclusion. The mortality rate for men was 21%, compared to 5% for women. This increased risk in men was independent of other variables often associated with mortality, such as age, ethnicity, obesity, alcohol consumption, smoking, sleep-disordered breathing, or depression [38].

When examining sex differences in circadian rhythm disorders, we found that women’s rest/activity patterns are more stable and more regular than men’s [23]. This conclusion is supported by another study, in which, according to circadian parameters (including intradaily variability (IV), interdaily stability (IS), amplitude, and I < O), women’s rhythm was more stable and less fragmented than men’s [39]. Moreover, other authors reported that from the age of 20 to 50 years, men tend to have an eveningness chronotype more frequently, whereas women present a morning chronotype more frequently. These sex differences seem to even out over time, starting from menopause in women [40]. In the study included in our review, the mean age was 49 years; thus, the lack of significant sex differences in chronotype is in line with the findings of the previously mentioned study.

We could identify only one risk factor related to circadian rhythm—shift work—highlighting the need for future studies exploring sex differences in the aetiology of circadian rhythm disorders. Irregular working hours alter sleep and wake times, eventually leading to circadian rhythm disturbances [41]. In one of the articles we examined, no gender difference was found regarding the relationship between shift work and circadian rhythm disorders [24]. However, another study reported that men adapt better than women to circadian rhythm fluctuations during shift work [42]. Given that shift work has increased over the past decade, and some previous findings are not in line with results of our review, future research on sex differences in circadian rhythm disorders is particularly important [43].

One of the strengths of this review is the rigorous and systematic approach, applying PRISMA requirements at all stages. Also, the review had no language restrictions. However, there are some important limitations. First, we chose a 5-year period because we aimed to provide up-to-date information, but the number of studies suffered as a result.

Second, the studies were heterogeneous, with most of them including different methods for assessing insomnia and circadian rhythm disorders, and therefore did not allow for more uniform analysis tools. A third limitation is that the data on sex differences that we sought to highlight were secondary in some studies. This created difficulties in extracting and analyzing the results. Moreover, only sex differences were reported, and no study investigated sex differences. We acknowledge that information about sex started to be collected very recently; thus, this aspect will likely be included in future studies. Nevertheless, we highlight here the need for considering sex aspects when evaluating sleep disorders in general, and insomnia and circadian rhythm disorders in particular. Fourth, most of the studies used subjective, self-reported questionnaires for data collection. Although this approach may be subject to bias, especially in observational and population-based studies, it is still appropriate and allows for the collection of data on large sample sizes.

## 5. Conclusions

In conclusion, our systematic review provided a comprehensive examination of the recent literature on sex differences in insomnia and circadian rhythm disorders. A synthesis of studies published in the past five years revealed that the prevalence of insomnia is higher in women than men. However, men suffer more from the long-term consequences of insomnia. The circadian rhythm is more regular in women. Studies show that insomnia and circadian rhythm disorder risk factors have varying effects on men and women. These factors include cerebrovascular and cardiometabolic influences, shift work, and infections. Healthcare professionals should consider sex differences and provide more individualized treatment, along with prevention strategies. In summarizing and interpreting the available evidence, we underline the importance of further research to promote a deeper understanding of the interaction between sex and sleep.

## Figures and Tables

**Table 1:** Summary of main findings for selected studies investigating sex differences in insomnia and circadian rhythm disorders.

Author(s), Year	Country	Study Design	Aims	Study Population	Main Findings
Höglund et al., 2020 [14]	Sweden	CSS ^1^	To compare different types of mental ill-health across age and sex groups with respect to symptom severity. To determine the prevalence of caseness of different types of mental ill-health in combinations of age groups and sex in both absolute and relative terms.	*N* = 3046 (females—1898, males—1508)	Women, in general, presented a higher risk for insomnia than men, with the highest risk for younger women and a lower risk for older women. For men, the highest risk for insomnia was in the 30–39 year age group. Insomnia symptom severity was higher in men than in women in the age group of 30–39 years and was lower in both men and women in those aged 50–69 years.
Salfi et al., 2020 [15]	Italy	LS ^2^	Investigate the changes in sleep and mental health changes during the prolonged lockdown due to the COVID-19 outbreak.	*N* = 2701 (females—2210, males—491)	Women seemed to be more resilient than men in the long-run, exhibiting a slight trend toward improvement in insomnia, depression, anxiety, and distress at the end of the seven weeks covered by the present research. On the other hand, men showed an exacerbation of insomnia symptoms and a deterioration of sleep quality during the lockdown. Furthermore, male participants reported a substantial increment of perceived stress at the end of the study. In addition, although women reported a higher prevalence of clinical conditions, such as insomnia and depression, in the first part of the lockdown, the sex gap was narrowed after four weeks.
Liu et al., 2021 [16]	China	CSS	To estimate the prevalence of insomnia among the general population during the COVID-19 outbreak. To examine the combined effect of sex and age on insomnia. To figure out the shared factors and the specific factors that are associated with insomnia.	*N* = 2858 (females—1532, males—1326)	There are no significant differences between sex and age in the prevalence of insomnia. Among all 2858 participants, 19.6% are found to have insomnia.
de Almondes et al., 2022 [17]	Greece	CSS	To evaluate the prevalence of sleep disorders in older adults, to describe their sleep patterns, as well as to analyze if there were any changes, in comparison with the pre-pandemic period.	*N* = 914 (females—372, males—542)	The COVID-19-related lockdown altered sleep habits and worsened sleep disorders among older adults. Sex comparisons were also made considering the worsening of each pre-existent sleep disorder. Significant changes were present in insomnia and restless leg syndrome in women and in sleep apnea in men. Women exhibited higher sleep latency, higher frequency of awakenings during sleep, lower sleep efficiency, lower sleep quality, and lower sleep awakening quality when compared to men.
Jeon et al., 2023 [18]	Korea	CSS	To assess perceived changes in the prevalence of insomnia due to the pandemic in in Korea and identify the factors associated with sleep changes during the pandemic outbreak.	*N* = 4000 (females—1965, males—2035)	The prevalence of insomnia was significantly higher in women than in men across all age groups. Insomnia prevalence was associated with female sex, night workers, being unmarried, and smoking. Among men, the prevalence was the highest in those aged 20–29 years, while among women, it was the highest in those aged 30–39 years and showed a decreasing tendency with age. Moderate to severe insomnia was reported by 12.9% of the participants. Among men, the 20–29 years age group had the highest prevalence, whereas among women, the 60–69 years age group had the highest prevalence.
Matas et al., 2022 [19]	Portugal	CS ^3^	Identify the features of patients who develop insomnia after an ischemic stroke and characterize them.	*N* = 157 (females—81, males—74)	Male sex and previous minor vascular events were significantly associated with the development of insomnia after ischemic stroke.
Silva-Costa et al., 2022 [20]	Brazil	CSS	Evaluate sex-specific associations between sleep problems and cardiometabolic risk factors.	*N* = 13,723 (females—7491, males—6232)	Obesity, hypertension, and high glycated hemoglobin were associated with self-reported sleep duration and insomnia symptoms (either separately or linked to short sleep duration) in women, but not in men. Cardiometabolic risk factors were associated with insomnia symptoms plus short sleep duration only in women. In relation to hypertriglyceridemia, statistically significant associations with insomnia symptoms were observed among both women and men.
Pudlo et al., 2022 [21]	Poland	CS	To assess the prevalence of insomnia in the early post-COVID-19 recovery period and explore differences in the results acquired from the Athens Insomnia Scale by sex and selected infection severity parameters.	*N* = 200 (females—99, males—101)	Women achieved a higher score in overall AIS. The analysis of the results obtained by all participants in the AIS shows a significant correlation with the duration of symptoms (days). The results suggest a higher risk of insomnia among women.
Huang et al., 2023 [22]	Australia	CS	Estimate the differences in CVD-free life expectancy between people with different sleep profiles.	*N* = 308,683 (females—173,546, males—135,137)	The study observed a gradual loss in CVD-free life expectancy toward poor sleep. Compared with healthy sleepers, female and male poor sleepers lost 1.80 and 2.31 CVD-free years, respectively, while female and male intermediate sleepers lost 0.48 and 0.55 years, respectively. Among men, those with clinical insomnia or sleep-related breathing disorders lost CVD-free life by 3.84 or 6.73 years, respectively. Among women, sleep-related breathing disorders or other sleep disorders were associated with 7.32 or 1.43 years lost, respectively.
Li et al., 2021 [23]	USA	CSS	To describe rest-activity rhythm (RAR) patterns among general adults and to explore variations by sex, age, and race/ethnicity.	*N* = 8200 (females—4224, males—3976)	Women had higher RAR amplitude and mesor and also more robust (pseudo-F statistic), more stable (higher interdaily stability), and less fragmented (lower intradaily variability) RAR than men. Women were also more likely have a normal acrophase than men.
Rodríguez-González-moro et al., 2020 [24]	Spain	CSS	To determine the prevalence of self-reported sleep quality and to investigate those factors that may predict the risk of suffering poor sleep quality in a large sample of public workers from Murcia.	*N* = 476 (females—232, males—244)	No significant differences were found according to sex in the overall sleep quality scores, but there were differences in the duration of sleep. The mean score in the reduced scale of the Morningness–Eveningness Questionnaire was similar between females and males. According to the scale, 62.0% of public workers had morning chronotypes, and 38.0% were classified as having an evening-intermediate chronotype. Fixed morning shifts and evening chronotypes were independent predictors of suffering from poor sleep quality.

^1^ Cross-sectional study; ^2^ longitudinal study; ^3^ cohort study.

## Data Availability

This systematic review involved the analysis and synthesis of data from previously published studies. All included studies and their respective data sources are clearly referenced in the manuscript.
